# Preanalytical Stability of 13 Antibiotics in Biological Samples: A Crucial Factor for Therapeutic Drug Monitoring

**DOI:** 10.3390/antibiotics13070675

**Published:** 2024-07-20

**Authors:** Paolo Dalla Zuanna, Debora Curci, Marianna Lucafò, Riccardo Addobbati, Antonella Fabretto, Gabriele Stocco

**Affiliations:** 1 Clinical and Experimental Pharmacology, Centro di Riferimento Oncologico di Aviano (CRO) IRCCS, 33081 Aviano, Italy; paolo.dallazuanna@studenti.units.it; 2 Institute for Maternal and Child Health IRCCS Burlo Garofolo, 34137 Trieste, Italy; debora.curci@burlo.trieste.it (D.C.); riccardo.addobbati@burlo.trieste.it (R.A.); antonella.fabretto@burlo.trieste.it (A.F.); 3 Department of Life Science, University of Trieste, 34127 Trieste, Italy; mlucafo@units.it; 4 Department of Medical, Surgical and Health Sciences, University of Trieste, 34149 Trieste, Italy

**Keywords:** antibiotics, stability, therapeutic drug monitoring, blood, plasma, serum, drugs

## Abstract

The stability of antibiotic preanalytical samples is a critical factor in therapeutic drug monitoring (TDM), a practice of undoubted importance for the proper therapeutic use of antibiotics, especially in complex management patients, such as pediatrics. This review aims to analyze the data in the literature regarding the preanalytical stability of some of the antibiotics for which TDM is most frequently requested. The literature regarding the preanalytical stability of amikacin, ampicillin, cefepime, ceftazidime, ciprofloxacin, daptomycin, gentamicin, levofloxacin, linezolid, meropenem, piperacillin, teicoplanin, and vancomycin in plasma, serum, whole blood, and dried blood/plasma spot samples was analyzed. Various storage temperatures (room temperature, 4 °C, −20 °C, and −80 °C) and various storage times (from 1 h up to 12 months) as well as subjecting to multiple freeze–thaw cycles were considered. The collected data showed that the non-beta-lactam antibiotics analyzed were generally stable under the normal storage conditions used in analytical laboratories. Beta-lactam antibiotics have more pronounced instability, particularly meropenem, piperacillin, cefepime, and ceftazidime. For this class of antibiotics, we suggest that storage at room temperature should be limited to a maximum of 4 h, storage at 2–8 °C should be limited to a maximum of 24 h, and storage at −20 °C should be limited to a maximum of 7 days; while, for longer storage, freezing at −80 °C is suggested.

## 1. Introduction

In the era of precision medicine, therapeutic drug monitoring (TDM) is an established clinical practice. Especially for certain drug classes, such as antibiotics, TDM proves to be essential for their correct use. In particular, TDM is important to ascertain the achievement of therapeutic concentrations and to avoid concentration-dependent toxicities. Some antibiotics, specifically, possess high inter-patient variability and, in some cases (e.g., aminoglycosides), have a low therapeutic index. For antibiotics, the practice of TDM is fundamental, also to avoid the emergence of antibiotic resistance and for their use in complex populations, such as pediatric or elderly patients, patients of different ethnicities, or the severely obese [1,2,3,4]. Furthermore, there is evidence that, in terms of cost-benefit, the practice of TDM is significantly beneficial [5].

In this context, knowledge of drug stability in biological samples is of paramount importance so that the TDM analysis does not turn out to be incorrect. All analytical laboratories should be aware of the stability data of each drug under analysis, under the various storage conditions, and for the various types of biological samples normally analyzed. In addition, many laboratories perform TDM analyses for distant hospitals; in this case, knowledge of the stability condition of each drug also determines the transport conditions that must be applied prior to arrival at the laboratory [6,7,8]. Incorrect evaluation or application of the stability data can lead to serious errors in the clinical treatment of patients. In particular, a potentially toxic drug concentration may not be recognized, or a correct drug concentration may be considered to be below the therapeutic range. Illustrative examples of the stability of beta-lactams are those described by Bahmany et al., who underline the fast drug degradation by hydrolysis of the beta-lactam ring [9], and by Kipper and collaborators, who found a bias in clearance and volume of distribution values of 30% and 28% after TDM of 5 beta-lactam antibiotics immediately after sampling and between 4 and 24 h of storage at room temperature [10]. Knowing the stability of a particular drug in biological samples is also important in deciding whether to modify the storage conditions with techniques such as changing the pH, derivatization, adding an enzyme inhibitor, or adding an antioxidant [7]. For example, the anti-Parkinson’s drug levodopa can be stored in plasma longer through the addition of the antioxidant sodium metabisulfite and the chelating agent ethylenediamine tetraacetic acid (EDTA) [11]; while for antibiotics, one example is the improved preservation of meropenem in aqueous solution by the addition of citrate buffer and the maintenance of pH values around 7 [12].

To analyze stability, it is first necessary to define, in numerical terms, what is meant by stability for drug concentrations (particularly drugs of the “small molecules” type) in human biological samples. According to the European Medicines Agency (EMA) guideline ICH guideline M10 on bioanalytical method validation and study sample analysis, effective 21 January 2023, for the concentration of a drug to be defined as stable, it must be within ±15% of the nominal concentration. In addition, regarding verification of stability after multiple freeze–thaw cycles, the guideline defines that at least three freeze–thaw cycles must be evaluated [13].

The scientific literature regarding reviews on the preanalytical stability of antibiotics in biological samples is rather poor. Since 2015, to our knowledge, only one review with these characteristics has been published, reporting the preanalytical stability of seven beta-lactam antibiotics (flucloxacillin, piperacillin, tazobactam, meropenem, cefalexin, cefazolin, and ceftazidime). This study generally observed instability after 6–12 h at room temperature, 2–3 days at 4 °C, 1–3 weeks at −20 °C, and good long-term stability at −70 °C [14]. Our work aims to revise and update the results of this review to January 2024 (only three antibiotics are in common with our chosen ones: piperacillin, meropenem, and ceftazidime) and implement the data with other antibiotics, including non-beta-lactams, for which TDM is nevertheless in high demand (aminoglycosides, quinolones, glycopeptides, lipopeptides, and oxazolidinones). Further, we will also consider stability with dried blood spot and dried plasma spot (DPS) sampling methods.

Therefore, the purpose of this paper is to provide an up-to-date review regarding the stability of 13 of the most common antibiotics for which TDM is required (amikacin, ampicillin, cefepime, ceftazidime, ciprofloxacin, daptomycin, gentamicin, levofloxacin, linezolid, meropenem, piperacillin, teicoplanin, and vancomycin), focusing on the different storage conditions (room temperature, 4 °C, −20 °C, −80 °C and subjecting it to multiple freeze–thaw cycles) and the different types of biological samples normally analyzed (plasma, serum, whole blood, and dried blood/plasma spot (DBS/DPS).

## 2. Results

In order to make the manuscript easier to read, the results are sorted alphabetically by drug name.

We analyzed 103 articles that included data on the pre-analytical stability of one or more of the thirteen antibiotics studied. Only a small proportion of the papers analyzed focused on the stability of the drug; the majority were papers validating the quantification methods of the antibiotics under study. This means that in most of the papers, a stability curve was not evaluated, but stability was only evaluated at certain time points. To overcome this problem, we have created graphs that are structured in such a way that, for each paper evaluated, the time period in which stability is guaranteed is shown in green, the time period in which stability was not evaluated is shown in grey and, finally, the time periods in which the drug was found as unstable are shown in red. If an article reports differences between plasma, serum, or whole blood preserves, these differences are reported in the text. In addition, the graphs indicate for each article whether it is a whole blood, plasma, or serum sample. If an article has results for more than one type of biological sample, the graph will separate the data and indicate the type of biological sample. Data on storage after three freeze–thaw cycles are not shown in the graphs but only in the text. Data on storage in DPS or DBS are treated separately at the end of each paragraph and are not shown in the graphs. If the stability of a drug has also been evaluated at temperatures other than the four standard temperatures (room temperature, 2–8 °C, −20 °C, and −80 °C), the data obtained are given separately in each paragraph and are not shown in the graphs, unless they are very similar to one of the standard temperatures (e.g., −70 °C has been merged with −80 °C and this difference has been clearly indicated in the graphs). When an article evaluated stability at different concentrations, if differences in stability were found, the concentration that degraded the most was included in the graphs, and this difference was clearly indicated in the text. All types of biological samples analyzed were human unless otherwise stated in the graphs.

### 2.1. Amikacin

**Figure 1:**
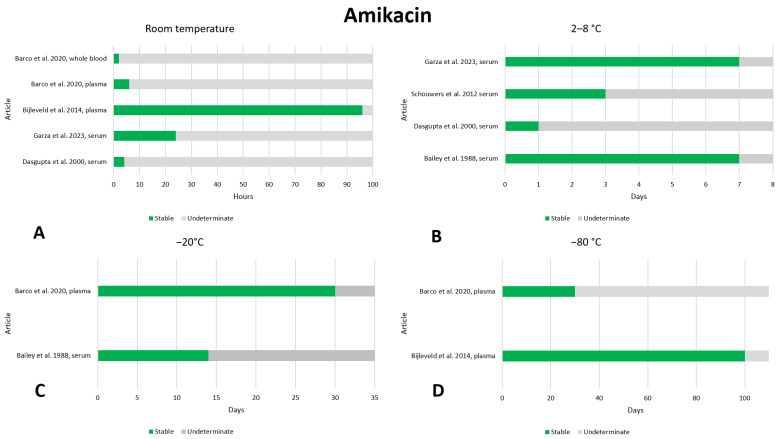
Graphical representation of the literature data regarding the preanalytical stability of amikacin. Only plasma, serum, and whole blood sampling are considered in the graphs. Data for storage at room temperature (**A**), 2–8 °C (**B**), −20 °C (**C**), and −80 °C (**D**) are shown. References include citations [15,17,18,19,20,21].

There are not many data on the stability of amikacin in the literature; we found seven articles that reported on the stability of amikacin [15,16,17,18,19,20,21]. The data are shown in Figure 1. No instability was found in any of these articles, except for storage in DPS at room temperature. At room temperature, amikacin was found to be stable after storage times of 4 h [17], 6 h [21], 24 h [19], and 4 days [20]; therefore, 4-day storage at room temperature can probably be considered safe. At the temperature of 2–8 °C, two articles evaluated stability after 7 days, noting that the drug was stable [15,19]. Other articles evaluated stability only after 24 h [17] or 3 days [18], and both found that the drug was stable; therefore, probably 7-day storage at 2–8 °C can be considered safe. Two articles evaluated stability at −20 °C for 14 [15] and 30 [21] days, finding that the drug was stable. At −80 °C one article assessed stability after 100 days, noting that the drug was stable [20]. Stability after three freeze–thaw cycles was evaluated in one work, and no instability was found [20].

In addition, one article evaluated the stability of amikacin after storage in DPS [16]. At 25 °C, the drug was stable for up to 2 days. At 2–8 °C, stability was evaluated for up to 14 days, and no instability was detected. Also, in DPS, amikacin was stable for 1 day after storage at 42 °C.

### 2.2. Ampicillin

**Figure 2:**
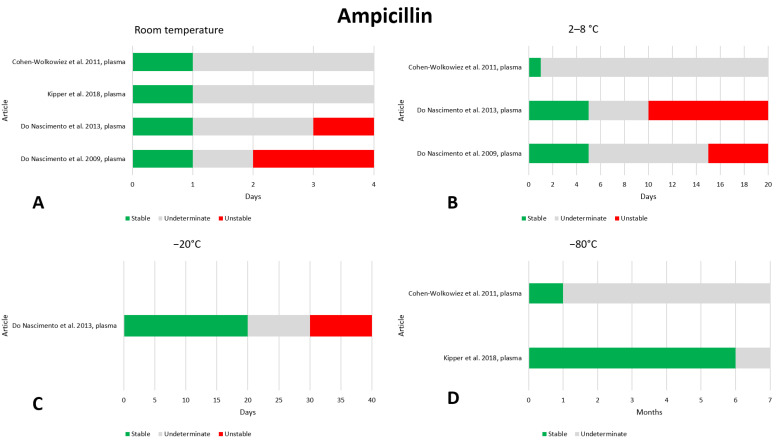
Graphical representation of the literature data regarding the preanalytical stability of ampicillin. Only plasma, serum, and whole blood sampling are considered in the graphs. Data for storage at room temperature (**A**), 2–8 °C (**B**), −20 °C (**C**), and −80 °C (**D**) are shown. References include citations [10,22,23,24].

We found four articles that reported on the preanalytical stability of ampicillin [10,22,23,24]. The data are shown in Figure 2. At room temperature, all articles agree that ampicillin is stable after 24 h; two articles evaluated stability at even later times after 2 [22] and 3 days [23], noting that the drug was not stable. These data suggest that ampicillin should not be stored at room temperature for more than 24 h. At temperatures of 2–8 °C most articles state that ampicillin remains stable for up to 5 days of storage; with longer storage, the stability is lost [22,23]. One article evaluated stability after storage at −20 °C and found that ampicillin is stable for up to 20 days, after which it becomes unstable [23]. Two articles evaluated stability at −80 °C, after storage for 1 month [24] and 6 months [10]; both found ampicillin to be stable. Finally, three studies evaluated the stability of ampicillin after three freeze–thaw cycles and found that the drug remained stable [10,23,24]. In any case, given the paucity of data on the preanalytical stability of ampicillin, we believe that it should be investigated further to confirm the above results.

### 2.3. Cefepime

**Figure 3:**
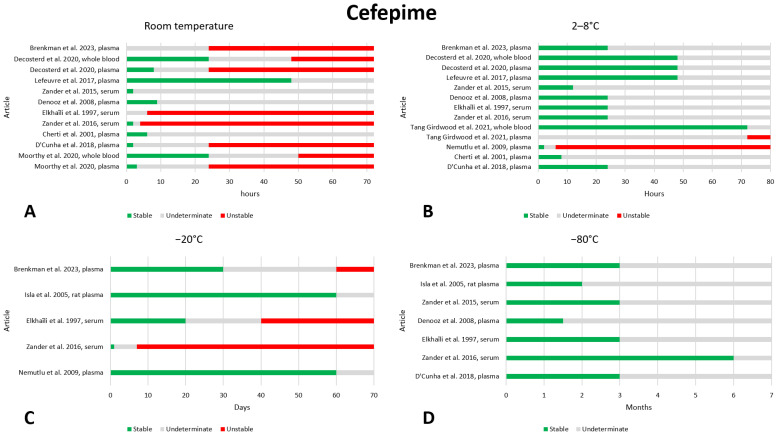
Graphical representation of the literature data regarding the preanalytical stability of cefepime. Only plasma, serum, and whole blood sampling are considered in the graphs. Data for storage at room temperature (**A**), 2–8 °C (**B**), −20 °C (**C**), and −80 °C (**D**) are shown. References include citations [25,26,27,28,29,30,31,32,33,34,35,36,37].

We found thirteen articles that reported on the preanalytical stability of cefepime [25,26,27,28,29,30,31,32,33,34,35,36,37]. The data are shown in Figure 3. With regard to stability at room temperature, the data in the literature are very variable, and it is difficult to draw conclusions. In most articles, in plasma, cefepime appears to be stable for at least 6 h [27,32,34,36], although two articles show instability at 4 [30] and 6 h [31], while one article reported stability even after 48 h of storage [34]. Two articles evaluated stability in both plasma and whole blood, clearly showing that cefepime has greater stability in whole blood at room temperature; in fact, after 24 h of storage, only the whole blood sample was found to be stable in both studies [25,36]. However, given the wide variability in the literature data, the stability of cefepime at room temperature should be further investigated. At 2–8 °C, the vast majority of articles show that cefepime is stable after 24 h of storage [26,30,31,32,34,36,37]; two of these articles also report good stability after 48 h of storage [34,36]. In contrast to these results, one article reports instability after 6 h of storage [28]. As observed for storage at room temperature, one article found greater stability in whole blood storage than in plasma after 72 h of storage [29]. When stored at −20 °C, most articles report that cefepime is stable after 20 days [28,31,35,37]; only one article found instability before 30 days, after only 7 days of storage [30]. No instability data has been found for storage at −80 °C, and most articles agree that a storage period of 3 months can be considered safe [26,30,31,33,37]. Stability after three freeze–thaw cycles was evaluated in seven works, and no instability was found [26,27,28,30,32,34,35].

In addition, one article evaluated the stability of cefepime after storage in DPS at room temperature and found that cefepime is certainly stable for 3 h, but after 24 h its stability is compromised [25].

### 2.4. Ceftazidime

**Figure 4:**
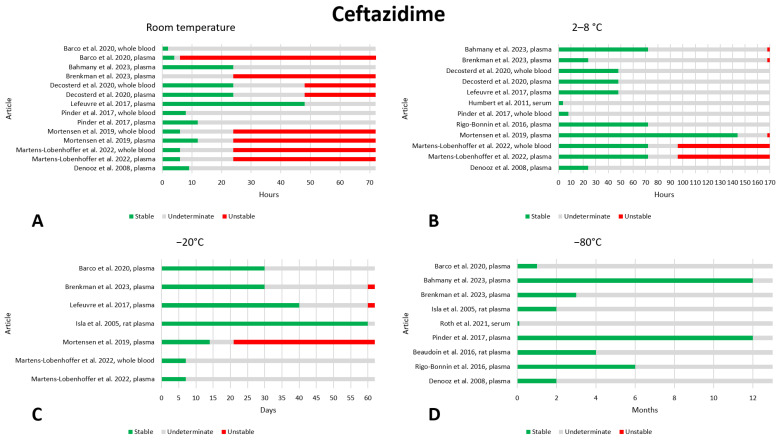
Graphical representation of data regarding the preanalytical stability of ceftazidime. Only plasma, serum, and whole blood sampling are considered in the graphs. Data for storage at room temperature (**A**), 2–8 °C (**B**), −20 °C (**C**), and −80 °C (**D**) are shown. References include citations [9,21,32,34,35,36,37,38,39,40,41,42,43,44].

Fourteen published articles included data on the preanalytical stability of ceftazidime [9,21,32,34,35,36,37,38,39,40,41,42,43,44]. The data are shown in Figure 4. With regard to storage at room temperature, after 12 h of storage, only one article noted that the drug was unstable [21] and five found stability [9,34,36,41,44]. On the other hand, after 24 h, four articles showed instability [21,37,38,44] and three noted that ceftazidime was stable [9,34,36]; therefore, we believe that storage at room temperature for 12 h can be considered safe. At 2–8 °C, the first instability was observed after 96 h of storage [38] and, in other articles, after 7 days [9,37,44]. All measurements taken at 48 [9,34,36,38,39,44] and 72 [9,38,39,44] h showed that ceftazidime was stable, suggesting no more than 72 h storage at 2–8 °C. At a temperature of −20 °C, four articles found that ceftazidime was stable after 30 [21,37], 40 [34], and even 60 [35] days. One article focusing on stability found that the drug was unstable after 21 days, which casts doubt on the above statement; we believe that stability at a storage temperature of −20 °C should be better investigated. With regard to freezing at −80 °C, no evidence of instability has been found, and based on the available data, we consider a storage period of twelve months to be safe [9,39,41]. Stability after three freeze–thaw cycles was evaluated in five works, and no instability was found [32,35,38,40,41].

In addition, one article evaluated the stability of ceftazidime after storage in DBS [38]. At room temperature, the drug is stable for 24 h, but stability is compromised after 48 h. Stability was assessed in DBS also at 2–8 °C after 7 days, and ceftazidime was found to be stable.

### 2.5. Ciprofloxacin

**Figure 5:**
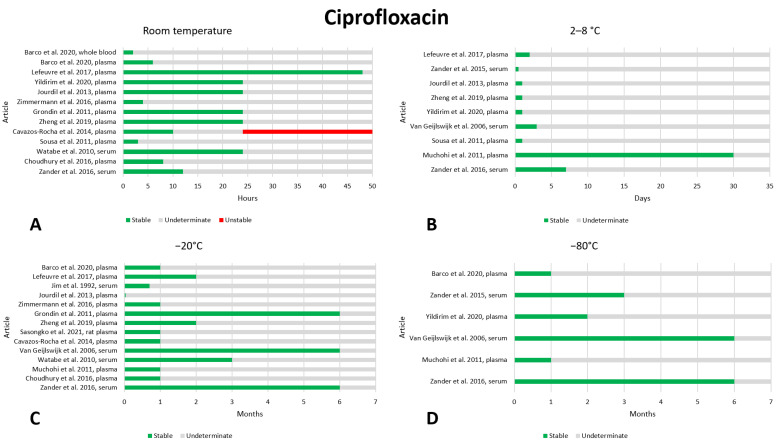
Graphical representation of data regarding the preanalytical stability of ciprofloxacin. Only plasma, serum, and whole blood sampling are considered in the graphs. Data for storage at room temperature (**A**), 2–8 °C (**B**), −20 °C (**C**), and −80 °C (**D**) are shown. References include citations [21,30,33,34,45,46,47,48,49,50,51,52,53,54,55,56,57].

We found seventeen articles that reported on the preanalytical stability of ciprofloxacin [21,30,33,34,45,46,47,48,49,50,51,52,53,54,55,56,57]. The data are shown in Figure 5. In general, ciprofloxacin appeared to have excellent stability under normal storage conditions. At room temperature, seven studies evaluated stability after 24 h of storage. Six of these studies found that the drug maintained adequate stability [33,34,47,53,54,56], while only one study found instability [50]. Based on these data, we believe that storage at room temperature for 24 h can be considered safe. For storage at 2–8 °C, most studies evaluated stability after a very short storage time (12–72 h), finding that the drug was stable; however, two studies evaluated stability after 7 days [30] and 30 days [46] and found the drug to be stable. It is, therefore, likely that ciprofloxacin is stable at 2–8 °C for longer than 72 h, but we believe that this needs to be further investigated. It should be noted that Muchohi et al. [46], who found stability for 30 days at a concentration of 1 mg/L, instead found instability after 7 days at a concentration of 3 mg/L; we excluded the higher concentration from the results because it showed that the drug was unstable after 7 days, even at −20 °C and −80 °C, temperatures at which ciprofloxacin is stable for many months. Therefore, we consider this to probably be an experimental error. No instability data were found for freezing at −20 °C; the time periods evaluated ranged from 1 month to 6 months. After 6 months, three studies [30,49,54] found the drug to be stable; therefore, we consider 6 months of storage at −20 °C to be safe. Similar to −20 °C, no instability data was found for freezing at −80 °C and the time periods evaluated ranged from 1 to 6 months [21,33,46,49,52]; therefore, we consider 6 months of storage at −80 °C to be safe despite the paucity of the available data. Stability after three freeze–thaw cycles was evaluated in eight works, and no instability was found [30,34,45,48,52,53,54,55].

### 2.6. Daptomycin

**Figure 6:**
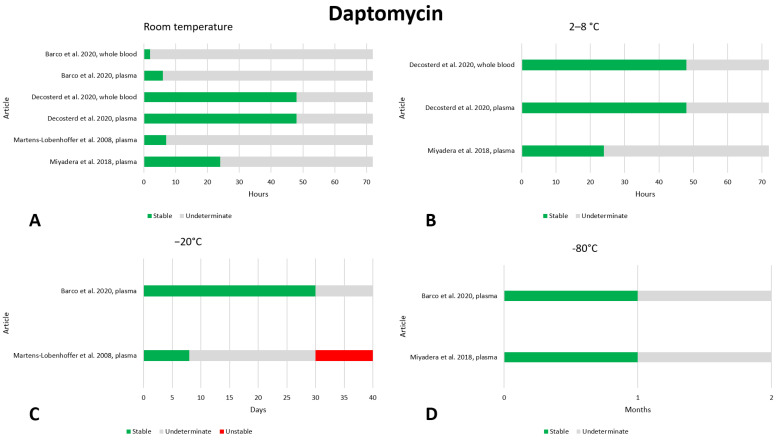
Graphical representation of data regarding the preanalytical stability of daptomycin. Only plasma, serum, and whole blood sampling are considered in the graphs. Data for storage at room temperature (**A**), 2–8 °C (**B**), −20 °C (**C**), and −80 °C (**D**) are shown. References include citations [21,36,59,61].

We found six articles that reported on the preanalytical stability of daptomycin [21,36,58,59,60,61]. The data are shown in Figure 6. Due to the limited data in the literature, it is difficult to define the preanalytical stability of daptomycin. At room temperature, daptomycin appears to be stable for at least 24–48 h [36,59], and the same applies to storage at 2–8 °C [36,59]; no data are available for longer storage at these temperatures. Two articles evaluated stability at a storage temperature of −20 °C and found different results. After 1 month of storage, one article found stability [21] and the other found instability [61]; therefore, we think that stability at −20 °C should be further evaluated. For storage at −80 °C, two studies evaluated the stability of daptomycin after 1 month, finding that the drug was stable [21,59]. Stability after three freeze–thaw cycles was evaluated in two works, and no instability was found [58,59].

In addition, one article evaluated the stability of daptomycin after storage in DPS [60]. At room temperature, the drug is stable for 7 days, but stability is compromised after 15 days. Stability was assessed in DPS also at 2–8 °C after 3 days, and daptomycin was found to be stable.

### 2.7. Gentamicin

**Figure 7:**
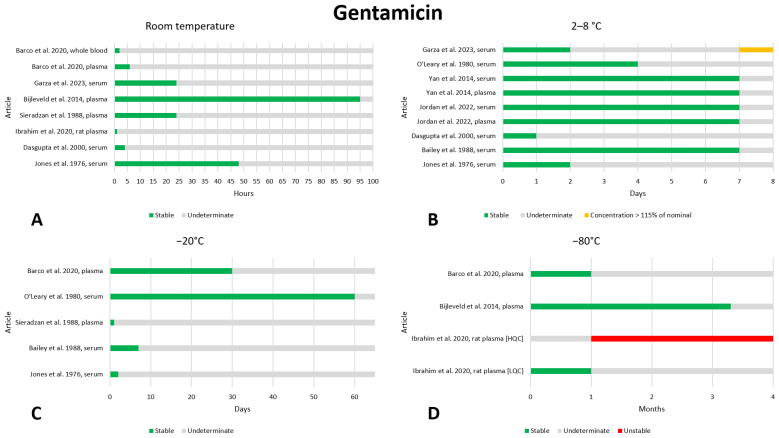
Graphical representation of data regarding the preanalytical stability of gentamicin. Only plasma, serum, and whole blood sampling are considered in the graphs. Data for storage at room temperature (**A**), 2–8 °C (**B**), −20 °C (**C**), and −80 °C (**D**) are shown. HQC = High-Quality Control, LQC = Low-Quality Control. References include citations [15,17,19,20,21,62,63,64,65,66,67].

Eleven published articles included data on the preanalytical stability of gentamicin [15,17,19,20,21,62,63,64,65,66,67]. The data are shown in Figure 7. Not many articles have evaluated the stability of gentamicin at room temperature, and most evaluated short storage periods from a few hours to 24 h [17,19,63,64], finding the drug to be stable. Two studies evaluated stability at room temperature for longer periods, finding stability even after 48 h [62] and after 96 h [20]. With regard to stability at 2–8 °C, the longest storage period evaluated was 7 days: three out of four articles found gentamicin to be stable [15,65,66], one article was found to exceed the nominal concentration [19]. In light of these data, storage for 7 days at 2–8 °C appears to be safe. Storage at −20 °C has been little studied. Only one old article evaluated stability after 2 months of storage and found the drug to be stable [67]; the other studies evaluated stability after one month [21], one week [15] or less [62,64] and always found stability. Storage at −80 °C was evaluated in three studies: Bijleveld et al. evaluated stability after 100 days and found that the gentamicin concentration was stable [20]; Barco et al. evaluated stability after 1 month and found that the gentamicin concentration was stable [21]; Ibrahim et al. demonstrated that after 1 month, gentamicin was stable in rat plasma at a high-quality control (HQC = 5 µg/L), while at a low-quality control (LQC = 1 µg/L), was unstable [63]. The latter study evaluated the stability of gentamicin in rat plasma even after three cycles of freezing and thawing and found that the drug was stable at a concentration of 5 µg/L but unstable at a concentration of 1 µg/L [63]. Except for storage at 2–8 °C. In light of these conflicting or insufficient data, we believe that the stability of gentamicin needs to be further investigated.

### 2.8. Levofloxacin

**Figure 8:**
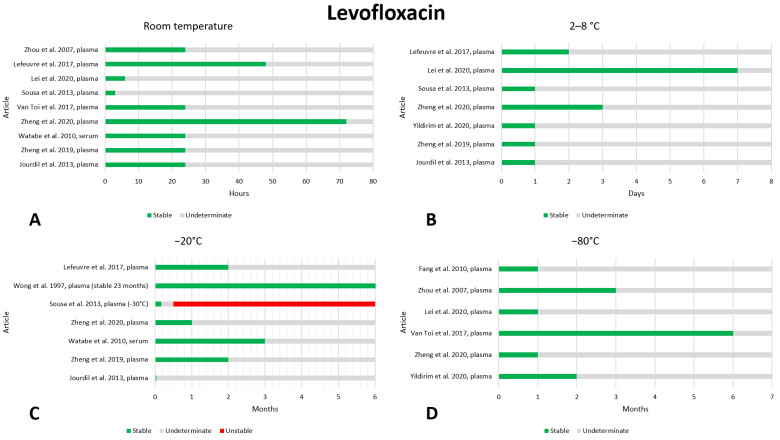
Graphical representation of data regarding the preanalytical stability of levofloxacin. Only plasma, serum, and whole blood sampling are considered in the graphs. Data for storage at room temperature (**A**), 2–8 °C (**B**), −20 °C (**C**), and −80 °C (**D**) are shown. References include citations [34,47,52,53,56,68,69,70,71,72,73,74].

We found twelve articles that reported on the preanalytical stability of levofloxacin [34,47,52,53,56,68,69,70,71,72,73,74]. The data are shown in Figure 8. Considering storage at room temperature, no instability has been detected in the timeframes assessed. Many studies evaluated stability after 24 h and found that levofloxacin was stable [34,47,53,56,68,69,73]. Two of these articles also evaluated stability over longer periods and found that the drug was stable at room temperature even after storage for 48 [34] and 72 [68] hours. With regard to stability at 2–8 °C, only one article evaluated stability after one week, finding that the drug was stable [72]; the other studies evaluated stability after shorter timeframes (24, 48, and 72 h), always finding proper stability [34,52,53,56,68,70]. For freezing at −20 °C, four articles state that levofloxacin is stable after 2 months of storage [34,47,53,71]; two of these also evaluate storage over longer periods, stating that the drug is stable after 3 months [47] and after 23 months [71]. In contrast to these data, Sousa et al. state stability after 5 days but state that the drug is not stable after 15 days, storing the sample at −30 °C [70]. With regard to freezing at −80 °C, no instability data were found; all studies consulted agreed that the drug maintained adequate stability after 1 month [52,68,69,72,73,74]. Three of these studies evaluated stability over longer periods, noting stability even after 2 months [52], 3 months [73] and 6 months [69], suggesting excellent stability of levofloxacin for long-term storage at a −80 °C freezing temperature. Stability after three freeze–thaw cycles was evaluated in ten works, and no instability was found [34,47,52,53,68,69,70,71,73,74].

### 2.9. Linezolid

**Figure 9:**
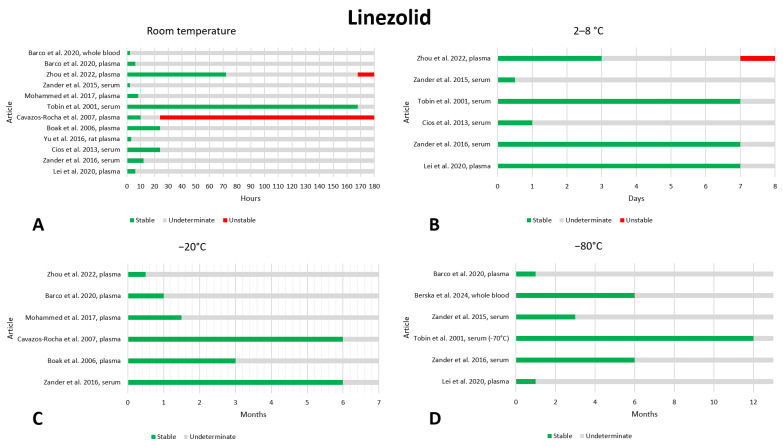
Graphical representation of data regarding the preanalytical stability of linezolid. Only plasma, serum, and whole blood sampling are considered in the graphs. Data for storage at room temperature (**A**), 2–8 °C (**B**), −20 °C (**C**), and −80 °C (**D**) are shown. References include citations [21,30,33,72,75,78,80,81,82,83,84,85].

Eighteen studies report on the preanalytical stability of linezolid in biological samples [21,30,33,60,72,75,76,77,78,79,80,81,82,83,84,85]. The data are shown in Figure 9. At room temperature, most articles found linezolid to be stable for the time periods studied, particularly after storage of 8 h [30,75,78,81,82,83] or less, 12 h [30,75,78,81,82], and 24 h [78,81,82,85]. In contrast, one study found linezolid to be stable after 10 h of storage at room temperature but unstable after 24 h [75]; other studies assessed stability after 3 days [85] and 7 days [82] and found stability. We believe that at room temperature, for storage times longer than 24 h, the stability of linezolid should be further investigated. For refrigeration at 2–8 °C, four studies evaluated the stability of linezolid after 7 days of storage; three found the drug to be stable [30,72,82] but one found instability [85]. The other studies evaluated much shorter storage times and always found stability [33,78]. At −20 °C, the longest storage time evaluated was 6 months. Two papers found linezolid to be stable [30,75] and other papers evaluated storage for shorter periods and found excellent stability. Similarly, at −80 °C, three articles found stability after 6 months of storage [30,82,84]; one of these went as far as evaluating stability after 12 months, also finding that linezolid was stable [82]. Two studies also examined stability at a storage temperature of −30 °C and found that linezolid was stable after storage times of 1 month [77] and 1.5 months [78]. Stability after three freeze–thaw cycles was evaluated in five works, and no instability was found [30,77,78,81,83].

Two articles also evaluated the stability of linezolid after storage in DBS [76,79]. Vu et al. evaluated stability at 50 °C for 7 days and found that the drug was stable [76]. Both studies investigated stability at 37 °C and at room temperature for 1 month and found good stability. La Marca et al. also evaluated stability for one-month storage at 2–8 °C and −20 °C, finding excellent stability [79].

In addition, one article evaluated the stability of linezolid after storage in DPS [60]. At room temperature, linezolid was found to be stable after 30 days of storage (except for the highest concentration of the three evaluated, where 18% degradation was detected). Finally, at a temperature of 2–8 °C, the drug showed good stability after 30 days of storage.

### 2.10. Meropenem

**Figure 10:**
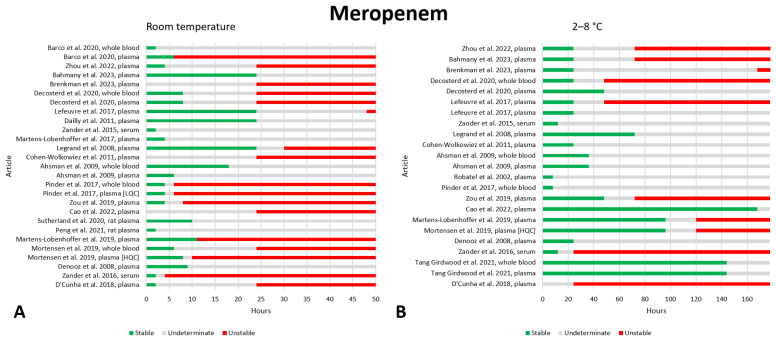

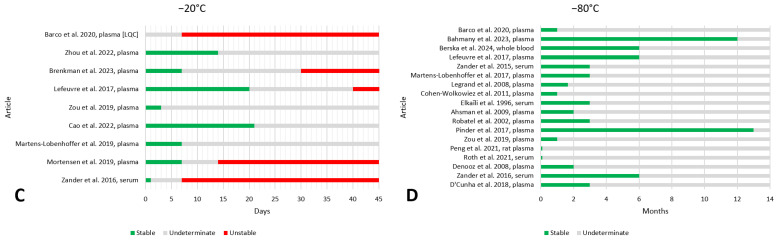
Graphical representation of data regarding the preanalytical stability of meropenem. Only plasma, serum, and whole blood sampling are considered in the graphs. Data for storage at room temperature (**A**), 2–8 °C (**B**), −20 °C (**C**), and −80 °C (**D**) are shown. LQC = Low-Quality Control, HQC = High-Quality Control. References include citations [9,21,24,26,30,32,33,34,36,37,41,42,44,84,85,86,87,88,89,90,91,93,94,95,96,97].

We found a significant number of papers containing data on the preanalytical stability of meropenem, twenty-nine papers in total [9,21,24,26,29,30,32,33,34,36,37,41,42,44,84,85,86,87,88,89,90,91,92,93,94,95,96,97,98]. The data are shown in Figure 10. At room temperature, the results are highly variable. Eleven studies found stability after 6 h [9,21,32,34,36,44,88,93,94,96,97], while two studies found instability at the same storage time [30,41], and one of these two studies found meropenem to be unstable even after 4 h [30]. After 8 h of storage, the same eleven articles, except one [21], reported stability, while four studies reported instability [21,30,41,90]. Therefore, we believe that storage for 6 h is likely to be safe, and storage for 8 h should be further investigated. For completeness, we report that at 12 h of storage, five studies found stability [9,34,93,94,97], and six reported instability [21,30,41,44,90,96]. Finally, after 24 h, four articles reported stability [9,34,94,97] while twelve reported instability [21,24,26,30,36,37,41,44,85,89,90,96]. Additionally, two articles evaluated stability at different concentrations with conflicting results. In one study, the LQC was more stable (LQC = 0.2 mg/L; HQC = 75 mg/L) [44], while in another, the opposite was true (LQC = 5 mg/L; HQC = 100 mg/L) [41]. Regarding stability at 2–8 °C, most articles, fifteen, agree that meropenem is stable after 24 h of storage [9,24,29,32,34,36,37,44,85,89,90,93,94,96,97]; however, two articles report instability after 24 h [26,30]. At 48 h, the number of studies reporting instability increases to four [26,30,34,36], although several studies also show good stability at 48 [29,36,44,89,90,94,96], 72 [29,44,89,94,96], 96 [29,44,89,96], 144 [29,89], and 168 [89] h. Commenting on these data, it seems that 24 h of storage at 2–8 °C can be considered safe, while longer storage times should be further investigated due to their variability. At this storage temperature, one article also found differences between storage in plasma and whole blood, noting that plasma storage was more stable [36]. Another article found that samples with lower concentrations were more stable than those with higher concentrations (LQC = 0.2 mg/L; HQC = 75 mg/L) [44]. Regarding freezing at −20 °C, seven out of nine studies agree that there is good stability after 7 days [34,37,44,85,89,96], and two studies instead found instability [21,30]. At longer timescales, the results are variable. In fact, after 2 weeks, three studies reported good stability [34,85,89], whereas two other articles found meropenem to be unstable [21,30,44]. We therefore recommend that storage under these conditions should not exceed 7 days. Additionally, one paper found differences in the stability of different concentrations of meropenem, with lower stability in LQC (LQC = 3 mg/L; HQC = 80 mg/L) [21]. No instability data were found for freezing at −80 °C; several articles evaluated stability up to 3 months [9,26,30,33,41,84,86,91,95,97] and five others up to 6 months [9,30,41,84,97]. According to the data presented, a storage period of 3 months seems to be a safe period. On the other hand, a storage period of 6 months at −80 °C can probably still be considered safe despite the paucity of available data. Stability after three freeze–thaw cycles was evaluated in twelve works, and no instability was found [24,26,30,32,41,86,87,88,90,91,92,94].

Two articles evaluated the stability of meropenem in DBS [96,98]. At room temperature, it seems that storage should not exceed 2 days, and at 2–8 °C, 7 days of storage should not be exceeded. Freezing at −20 °C should not exceed 14 days. Stability at 40 °C was also evaluated, noting that storage time should not exceed 12 h [98]. Stability at −40 °C for 30 days was also evaluated, and meropenem was found to be stable [98].

Finally, one article evaluated the stability of meropenem in DPS [89]. At 40 °C, stability was compromised after 24 h. At room temperature, stability is guaranteed for 24 h but not for 48 h. Refrigeration at 2–8 °C was evaluated for 1 week, and meropenem was stable, while freezing at −20 °C was evaluated for 3 weeks, and stability was demonstrated.

### 2.11. Piperacillin

**Figure 11:**
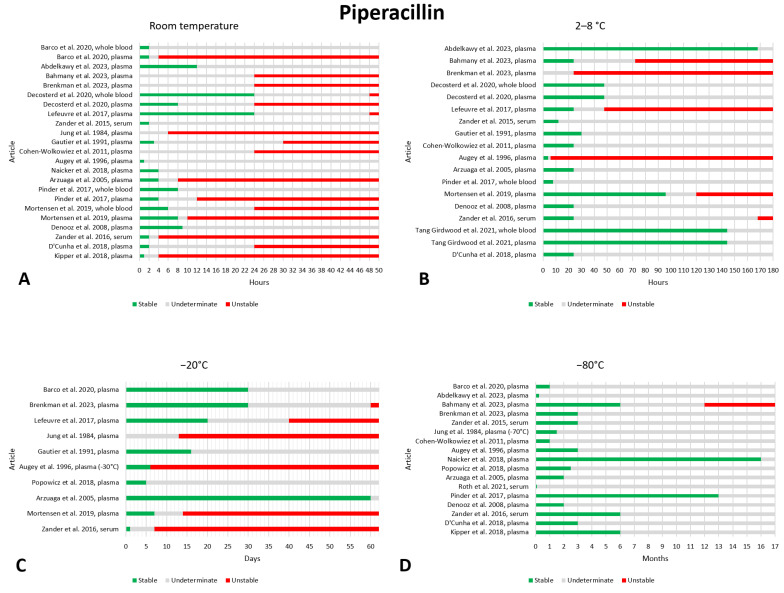
Graphical representation of data regarding the preanalytical stability of piperacillin. Only plasma, serum, and whole blood sampling are considered in the graphs. Data for storage at room temperature (**A**), 2–8 °C (**B**), −20 °C (**C**), and −80 °C (**D**) are shown. HQC = High-Quality Control. References include citations [9,10,21,24,26,29,30,32,33,34,36,37,41,42,44,99,100,102,103,104,105,106].

We found twenty-three papers containing data on the preanalytical stability of piperacillin [9,10,21,24,26,29,30,32,33,34,36,37,41,42,44,99,100,101,102,103,104,105,106]. The data are shown in Figure 11. Several articles have evaluated stability at room temperature. Looking at the data, it appears that stability is guaranteed for 4 h storage, at which time eight papers show piperacillin to be stable [32,34,36,41,44,99,102,106] despite three reports of instability [10,21,30]. At 6 h storage, six papers report stability [32,34,36,41,44,106] while four report instability [10,21,30,105]. After 8 h storage, six papers reported stability [32,34,36,41,44,106] while five found piperacillin to be unstable [10,21,30,99,105]. Based on these data, a maximum storage time of 4 h appears to be safer than 6 h. One paper also found differences between room temperature storage in plasma and whole blood, with whole blood being more stable [36]. Finally, two papers evaluated stability at different concentrations with conflicting results: in one study, the LQC was more stable (LQC = 0.5 mg/L; HQC = 150 mg/L) [10] while in another the opposite was true (LQC = 6 mg/L; HQC = 160 mg/L) [21]. When considering storage at 2–8 °C, most articles, twelve, agree that piperacillin is stable after 24 h of storage [9,24,26,29,30,32,34,36,44,99,104,106], only two articles reported instability [37,103]. After 48 h, four articles found stability [29,36,44,106], while three found piperacillin to be unstable [34,37,103]; therefore, it appears that 24 h storage at 2–8 °C can be considered safe, whereas 48 h cannot. It should also be noted that one article found the drug to be unstable after 6 h [103], while two papers found piperacillin to be stable even after 6 days [29,106], showing the high variability of these measurements. When it comes to storage at −20 °C, the data obtained from the literature analysis are quite variable. After one week of storage, six papers reported that piperacillin was stable [21,34,37,44,99,104], while two found instability [30,103]. However, when considering a storage period of 5 days, no instability data were found, while eight papers confirmed the good stability of piperacillin [34,37,44,99,100,101,103,104]. Probably, a storage period of 7 days could be considered quite safe, but on the basis of our research, it seems safer not to exceed a storage period of 5 days. After 2 weeks, almost half of the papers reported instability, suggesting the unreliability of this storage schedule. Finally, with regard to storage at −80 °C, the only finding of instability in the seventeen papers reviewed was after 12 months of storage [9]. After 3 and 6 months of storage, piperacillin appears to be stable, as confirmed by nine [9,10,26,30,33,37,41,102,103] and five [9,10,30,41,102] papers respectively. Stability after three freeze–thaw cycles was evaluated in ten works, and no instability was found [10,24,26,30,32,34,41,99,102,103].

Finally, one paper evaluated the stability of piperacillin in DBS [101]. At room temperature, stability was assessed after one week and found to be impaired; at −20 °C, stability was assessed after one month and it was found that the drug was stable. Good stability was also found after three freeze–thaw cycles.

### 2.12. Teicoplanin

**Figure 12:**
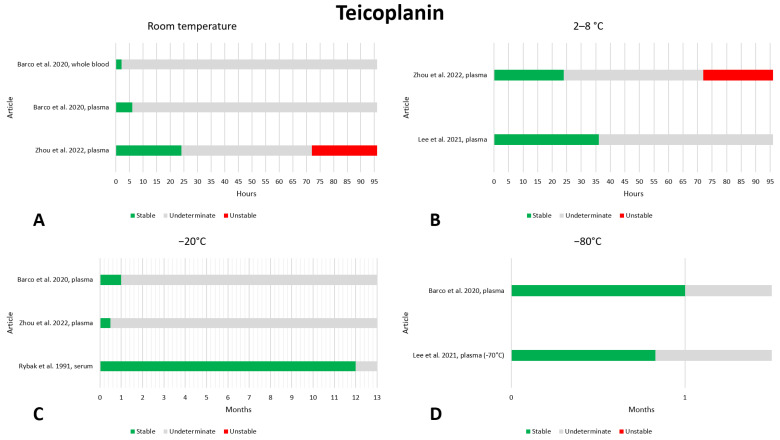
Graphical representation of data regarding the preanalytical stability of teicoplanin. Only plasma, serum, and whole blood sampling are considered in the graphs. Data for storage at room temperature (**A**), 2–8 °C (**B**), −20 °C (**C**), and −80 °C (**D**) are shown. References include citations [21,85,107,108].

We did not find much data on the preanalytical stability of teicoplanin; a total of four articles [21,85,107,108]. The data are shown in Figure 12. With regard to storage at room temperature, the data found were too limited to draw any conclusions; the only data found showed instability after 72 h of storage [85], whereas teicoplanin was stable after 6 [21,85] and 24 [85] hours. At 2–8 °C, the only available data show stability after 24 [85,108] and 36 [108] h, but instability after 72 h storage [85]. No evidence of instability was found for freezing at −20 °C. Assessments were made at 12 months [107], 1 month [21], and 2 weeks [85] and stability was always found to be within the acceptable ranges. The same can be said for freezing at −80 °C, where stability was assessed after 1 month [21] and 24 days [108] of storage. Stability after three freeze–thaw cycles was evaluated in one work, and no instability was found [108]. Given the paucity of data in the literature, we believe that the preanalytical stability of teicoplanin requires further investigation.

### 2.13. Vancomycin

**Figure 13:**
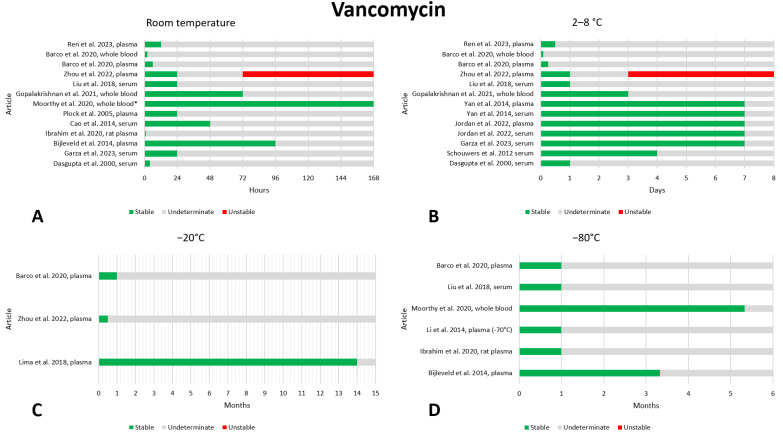
Graphical representation of data regarding the preanalytical stability of vancomycin. Only plasma, serum, and whole blood sampling are considered in the graphs. Data for storage at room temperature (**A**), 2–8 °C (**B**), −20 °C (**C**), and −80 °C (**D**) are shown. References include citations [17,18,19,20,21,63,65,66,85,109,110,111,112,113,114,115,116]. * Stability assessed up to 12 days, with no signs of degradation; stability up to 168 h is shown on the graph for better graphical visualization.

We found sixteen articles that reported on the preanalytical stability of vancomycin [17,18,19,20,21,63,65,66,85,89,109,110,111,112,113,114,115]. The data are shown in Figure 13. Regarding stability at room temperature, only one paper detected degradation of vancomycin after 72 h of storage [85]. In contrast, three papers found vancomycin to be stable after 72 h [20,111,113] and two of these, going up to 96 h, showed stability [20,111]. For greater confidence, we report that four papers found stability after 48 h [20,111,113,116] and eight papers found stability after 24 h of storage [19,20,85,110,111,113,114,116]. In addition, one paper evaluated stability at room temperature after 12 days and found vancomycin to be stable [111]. Even for storage at 2–8 °C, only one article found instability, the same as above, again after 3 days of storage, placing it as an outlier compared to all the others [85]. The other papers always found adequate stability, evaluated after 1 [17,18,19,65,66,85,113,114], 3 [18,19,65,66,113], or 7 days [19,65,66]. Only three articles evaluated the stability of vancomycin at −20 °C, always finding adequate stability; evaluations at this temperature were made after 14 months [112], 1 month [21] and 2 weeks [85]. Even for storage at −80 °C, no degradation data were found; most articles evaluated stability after 1 month [21,63,109,114], only two papers went further and evaluated degradation after 100 [20] and 160 [111] days. In addition, two papers evaluated stability at −40 °C and found that vancomycin was stable after storage for 7 days [115] and 2 months [116]. Stability after three freeze–thaw cycles was evaluated in five works, and no instability was found [63,112,114,115,116].

## 3. Discussion

Knowledge of the stability of drugs in blood samples is key to obtaining accurate measurements in TDM analyses, especially for therapies where TDM is essential, such as antibiotic therapies. Knowledge of the stability data allows plasma concentrations not to be underestimated, which could lead to poorer patient treatment and increased healthcare costs associated with adverse drug reactions. It is important to note that the maximum loss of 15% must include the entire process from sample collection to analysis. For example, if a sample is stored at room temperature for 2 h, refrigerated at 2–8 °C for 24 h, and then frozen at −20 °C for 1 week, the proportions lost in each of the three phases add up, and estimating the total loss may prove critical for a laboratory practicing TDM. This means that when several storage steps are added together, adherence to the accepted time frame for each temperature may not be sufficient to ensure stability.

Drugs can degrade in biological samples for a variety of reasons. There can be a metabolic type of instability characterized by enzymatic degradation of the molecules, or there can be a chemical instability, which usually occurs by oxidation, hydrolysis, or isomerization of the molecules under analysis. In addition, loss of analyte from samples can also occur by non-degradative methods, such as aggregation, precipitation, and non-covalent binding to tubing surfaces or to components of the biological fluid [117]. On the basis of their stability, the antibiotics studied in our work can be divided into two groups: beta-lactam antibiotics and non-beta-lactam antibiotics. The former is characterized by a much greater instability due to the opening of the beta-lactam ring by hydrolysis, which can be catalyzed by an enzyme, acid pH, the presence of ions, or the presence of nucleophilic agents [9,118].

Regarding beta-lactam antibiotics (ampicillin, cefepime, ceftazidime, meropenem, and piperacillin), stability is well defined precisely because, for the reasons given above, their degradation is much faster than that of the other antibiotics analyzed, and it is, therefore, possible, at the times evaluated, to clearly define when a drug is stable and when its stability is no longer guaranteed. Meropenem and piperacillin are the two most unstable drugs, and based on our data, storage at room temperature should not exceed 4 h for piperacillin and 6 h for meropenem, and for both should not exceed 24 h at 2–8 °C, 1 week at −20 °C, and 6 months at −80 °C. Cefepime must be stored for no longer than 6 h at room temperature, 24 h at 2–8 °C, and 20 days at −20 °C. Ceftazidime is more stable and requires storage for no longer than 12 h at room temperature and 72 h at 2–8 °C, while storage at −20 °C should be investigated further due to some conflicting data. The stability of the latter two drugs at −80 °C is comparable to that of meropenem and piperacillin. Ampicillin seems to be the most stable beta-lactam of the five studied, despite the limited data available in the literature; storage at room temperature should not exceed 24 h, at 2–8 °C 5 days, at −20 °C 20 days, and at −80 °C stability seems to be guaranteed for at least 6 months.

Based on these premises, we do not have much degradation data for the non-beta-lactam antibiotics (amikacin, ciprofloxacin, daptomycin, gentamicin, levofloxacin, linezolid, teicoplanin, and vancomycin) and can only confirm good stability at certain temperatures. To summarize, at room temperature, all these drugs appear to be sufficiently stable after 24 h storage; at 2–8 °C stability is confirmed for at least one week; at −20 °C stability appears to be guaranteed for at least one month, and at −80 °C stability has been confirmed after storage times of 6 months (ciprofloxacin, levofloxacin, and linezolid), 3 months (amikacin and vancomycin), and 1 month (daptomycin, gentamicin, and teicoplanin). Exceptions to this summary are the stability of daptomycin and teicoplanin at 2–8 °C (for which we have no data beyond 48 and 36 h, respectively) and the stability of daptomycin at −20 °C (for which data are scarce and contradictory [21,61]). Concerning the room temperature stability of ciprofloxacin and linezolid, the same author in two different works, [50] and [75], respectively, reports that the two drugs are stable for less time than reported in other works; therefore, these two outliers obtained from the same laboratory could be systematic errors. With regard to the stability of the fluoroquinolones ciprofloxacin and levofloxacin, these appear to be stable in almost all the contexts evaluated, except in two articles concerning the stability of ciprofloxacin at room temperature for more than 72 h [50] and the stability of levofloxacin at −30 °C for more than 30 days [35]. In these two cases, it is possible that stability was lost by the chelation of metal ions and formation of insoluble chelates, a known mechanism of instability of fluoroquinolones in aqueous solution [119], but further studies are needed.

We refer to the specific sections in the results chapter for a separate evaluation of these results. A summary of the time frame in which we consider guaranteed stability, based on the data obtained, is provided in Table 1.

Ten articles evaluated the stability of drugs in more than one of plasma, serum, and whole blood [21,25,29,36,38,41,44,65,66,93]; therefore, we could assess possible differences in the stability of drugs in different biological matrices. In general, no differences were observed except in a few cases. In particular, the stability in whole blood was better than in plasma for cefepime, both at room temperature [25,36] and at 2–8 °C [29], and for piperacillin at room temperature [36]. In contrast, one article showed that meropenem was more stable in plasma than in whole blood at room temperature [36]. It can be hypothesized that some drugs are more stable in whole blood samples and others in plasma. This hypothesis should be tested in further studies. Some mechanisms that may alter the stability of drugs between whole blood and plasma are the use of anticoagulants in whole blood, which may lead to a change in pH, the oxygen scavenging property of hemoglobin in whole blood, which may lead to less instability due to less oxidation, or the presence of erythrocyte enzymes in whole blood in addition to plasma enzymes, which may contribute to drug degradation [120].

A large number of articles have evaluated the stability of drugs at different concentrations, allowing the influence of drug concentration on the pre-analytical stability to be assessed. In general, no differences were observed except in a few cases. In particular, HQC was more stable than LQC in four articles: storage of gentamicin at −80 °C (5 vs. 1 µg/L) [63], meropenem at room temperature (100 vs. 5 mg/L) [41] and −20 °C (80 vs. 3 mg/L) [21], and piperacillin at room temperature (160 vs. 6 mg/L) [21]. In contrast, LQC was more stable than HQC in three cases on the stability of meropenem at room temperature and at 2–8 °C (0.2 vs. 75 mg/L) [44], and on the stability of piperacillin at room temperature (0.5 vs. 150 mg/L) [10]. Given the rarity of these observations and their contradictory results, it is likely that these are experimental errors.

We found ten articles containing data on the stability of the antibiotics studied in DPS or DBS [16,25,38,60,76,79,89,96,98,101]. In general, the stability of drugs in DPS/DBS was found to be better than storage in plasma, serum, or whole blood, suggesting that DPS/DBS sampling is a more effective method of sample storage. In an illustrative case study, storage of meropenem in DBS at room temperature appears to be guaranteed for up to 24–48 h [96,98], in contrast to storage in liquid samples, which should not exceed 6 h. In addition, five studies evaluated stability in DPS/DBS at high temperatures, between 37 °C and 50 °C, and found interesting results [16,76,79,89,98]. For example, the stability of meropenem at 40 °C seems to be guaranteed for up to 12 h [98]. This last consideration leads us to believe that sampling in DPS/DBS may be particularly useful in warmer countries or in summer seasons where transport to the laboratory under refrigerated or frozen conditions is not possible. It is an expected fact that many drugs are more stable in DBS/BPS than in whole blood, plasma, or serum because DPS and DBS samples are dried, which significantly reduces the water content of the sample. This dehydration minimizes hydrolytic degradation and degradation due to water-dependent enzymes [96,121,122,123].

Many of the articles consulted evaluated the stability of the antibiotics studied after three freeze–thaw cycles, and the result was that the drugs were stable in essentially all situations evaluated.

Finally, it should be noted that the data in the literature can only, in some cases, represent a true stability curve, giving precise information on the allowed storage times for each drug. In particular, it is only for drugs whose stability has been extensively studied and whose degradation is fairly rapid that it is possible to determine true storage times beyond which it is forbidden to extend storage, such as the beta-lactam antibiotics meropenem, piperacillin, cefepime, and ceftazidime. In other cases, stability has been less investigated, and as these drugs are mostly stable at normal storage times, articles often confirm stability at certain times but do not evaluate longer storage times, thus preventing a true instability figure from being obtained. A separate argument can be made for the beta-lactam antibiotic ampicillin, for which degradation data are available, but due to the paucity of results in the literature, we believe that the results should be confirmed with further studies.

## 4. Materials and Methods

A literature search was performed in the PubMed database. The research is updated to January 2024. The search terms included the following: (amikacin OR ampicillin OR cefepime OR ceftazidime OR ciprofloxacin OR daptomycin OR gentamicin OR levofloxacin OR linezolid OR meropenem OR piperacillin OR teicoplanin OR vancomycin) AND stability in blood. All abstracts were manually screened for the presence of an analytical method or any mention of stability. The full text of all selected journal articles was reviewed manually. To respect the European Medicines Agency (EMA) guideline ICH guideline M10 on bioanalytical method validation and study sample analysis [13], we excluded articles using stability criteria other than ± 15% of nominal concentration if the raw degradation data could not be obtained from the article or the Supplementary Materials. A flow chart concerning the articles found and those selected for each drug can be found in the Supplementary Materials.

## 5. Conclusions

In conclusion, we suggest that for non-beta-lactam antibiotics, storage at room temperature is guaranteed for at least 24 h, storage at 2–8 °C is guaranteed for at least 1 week, storage at −20 °C is guaranteed for at least 1 month, and storage at −80 °C is guaranteed for at least 6 months. For beta-lactam antibiotics, there is some variation in their stability, but to summarize and to be certain of stability, we suggest that storage at room temperature should not exceed 4 h, storage at 2–8 °C should not exceed 24 h, and storage at −20 °C should not exceed 1 week; whereas storage at −80 °C appears to be stable for at least 6 months. In addition, storage in whole blood may increase stability compared to plasma and serum, but due to the paucity of data, this aspect should be investigated further. Drug concentration does not appear to affect stability. Storage of DPS/DBS seems to be better than that of liquid samples. Finally, all drugs were stable after three freeze–thaw cycles.

Potential limits of this review are the use of only one database and the relatively small number of keywords used.

Ultimately, we believe that these data may prove useful in the correct execution of TDM analyses, minimizing errors and thus leading to better therapy management, particularly where TDM is essential, such as for complex patients or special populations like pediatric patients.

## Figures and Tables

**Table 1:** Summary of the pre-analytical stability of the 13 drugs analyzed. The table shows the storage times that we consider to be safe based on the data collected. Storage times where a drug is likely to be stable but where there is some doubt due to a lack of available data are shown in bold. Storage times at which a drug is likely to be stable but some doubt remains due to conflicting data are shown in red. Storage times at room temperature are given in hours, at 2–8 °C and −20 °C in days, and at −80 °C in months.

	Room Temperature (Hours)	2–8 °C (Days)	−20 °C (Days)	−80 °C (Months)
Amikacin	**96**	**7**	**30**	**3.3**
Ampicillin	24	5	**20**	1 (**6**)
Cefepime	6	1	30	3 (**6**)
Ceftazidime	12	3	30	12
Ciprofloxacin	24	7	180	180
Daptomycin	**48**	**2**	8 (**30**)	**1**
Gentamicin	48 (**96**)	7	30 (**60**)	1 (**3.3**)
Levofloxacin	24 (**72**)	2 (**7**)	60	3 (**6**)
Linezolid	24	7	180	6 (**12**)
Meropenem	6	1	7	6 (**12**)
Piperacillin	4	1	5 (7)	6
Teicoplanin	**24**	**1.5**	**365**	**1**
Vancomycin	96	7	30 (**420**)	1 (**5.3**)

## Data Availability

Raw data are available on PubMed database.

## Supplementary Materials

The following supporting information can be downloaded at: https://www.mdpi.com/article/10.3390/antibiotics13070675/s1.
