# Supplementary material for: Preanalytical Stability of 13 Antibiotics in Biological Samples: A Crucial Factor for Therapeutic Drug Monitoring
Source: Antibiotics (Basel). 2024 Jul 20;13(7):675. doi: 10.3390/antibiotics13070675 (PMC11274111; doi:10.3390/antibiotics13070675)
Supplement: Supplementary file 1 [file antibiotics-13-00675-s001.zip › antibiotics-3109938-supplementary.pptx]

## Slide 1
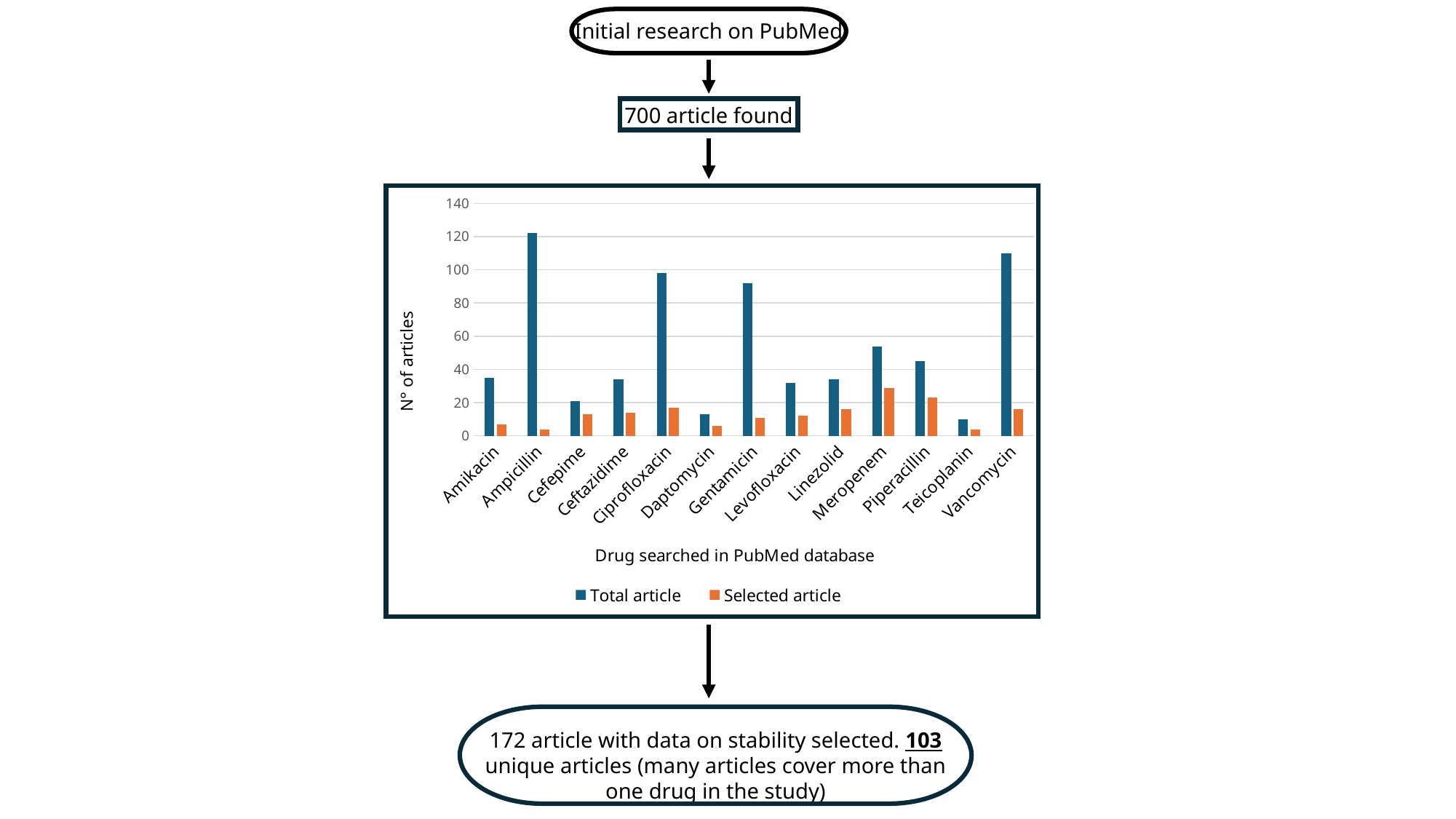

Initial research on PubMed
700 article found
### Chart
| Category | Total article | Selected article |
|---|---|---|
| Amikacin | 35.0 | 7.0 |
| Ampicillin | 122.0 | 4.0 |
| Cefepime | 21.0 | 13.0 |
| Ceftazidime | 34.0 | 14.0 |
| Ciprofloxacin | 98.0 | 17.0 |
| Daptomycin | 13.0 | 6.0 |
| Gentamicin | 92.0 | 11.0 |
| Levofloxacin | 32.0 | 12.0 |
| Linezolid | 34.0 | 16.0 |
| Meropenem | 54.0 | 29.0 |
| Piperacillin | 45.0 | 23.0 |
| Teicoplanin | 10.0 | 4.0 |
| Vancomycin | 110.0 | 16.0 |
172 article with data on stability selected. 103 unique articles (many articles cover more than one drug in the study)
